# Use of STAT6 Phosphorylation Inhibitor and Trimethylglycine as New Adjuvant Therapies for 5-Fluorouracil in Colitis-Associated Tumorigenesis

**DOI:** 10.3390/ijms21062130

**Published:** 2020-03-20

**Authors:** Mónica G. Mendoza-Rodríguez, C. Ángel Sánchez-Barrera, Blanca E. Callejas, Verónica García-Castillo, Diana L. Beristain-Terrazas, Norma L. Delgado-Buenrostro, Yolanda I. Chirino, Sonia A. León-Cabrera, Miriam Rodríguez-Sosa, Emma Bertha Gutierrez-Cirlos, Carlos Pérez-Plasencia, Felipe Vaca-Paniagua, Marco Antonio Meraz-Ríos, Luis I. Terrazas

**Affiliations:** 1 Unidad de Biomedicina, Facultad de Estudios Superiores Iztacala, Universidad Nacional Autónoma de México, Avenida de los Barrios 1, Los Reyes Iztacala, Tlalnepantla 54090, Mexico; monymendoza2704@gmail.com (M.G.M.-R.); angel.csb@hotmail.com (C.Á.S.-B.); aletse_bianca@hotmail.com (B.E.C.); garciaver@gmail.com (V.G.-C.); diana_laura510@hotmail.com (D.L.B.-T.); nlbuenrostro@gmail.com (N.L.D.-B.); irasemachirino@gmail.com (Y.I.C.); iicisabimendozamonica@hotmail.com (S.A.L.-C.); rodriguezm@unam.mx (M.R.-S.); carlos.pplas@gmail.com (C.P.-P.); felipe.vaca@gmail.com (F.V.-P.); 2 Instituto Nacional de Cancerología, Subdirección de Investigación Básica, Av. San Fernando No. 22, Ciudad de México 14080, Mexico; 3 Laboratorio Nacional en Salud, Facultad de Estudios Superiores Iztacala, Universidad Nacional Autónoma de Mexico, Avenida de los Barrios 1, Los Reyes Iztacala, Tlalnepantla 54090, Mexico; 4 Centro de Investigación y de Estudios Avanzados del Instituto Politécnico Nacional, Avenida IPN 2508, San Pedro Zacatenco, Ciudad de México 07360, Mexico; mmeraz@cinvestav.mx

**Keywords:** colorectal cancer, STAT6, Trimethylglycine, drug resistance, 5-FU, adjuvant therapies

## Abstract

Colorectal cancer (CRC) is one of the most widespread and deadly types of neoplasia around the world, where the inflammatory microenvironment has critical importance in the process of tumor growth, metastasis, and drug resistance. Despite its limited effectiveness, 5-fluorouracil (5-FU) is the main drug utilized for CRC treatment. The combination of 5-FU with other agents modestly increases its effectiveness in patients. Here, we evaluated the anti-inflammatory Trimethylglycine and the Signal transducer and activator of transcription (STAT6) inhibitor AS1517499, as possible adjuvants to 5-FU in already established cancers, using a model of colitis-associated colon cancer (CAC). We found that these adjuvant therapies induced a remarkable reduction of tumor growth when administrated together with 5-FU, correlating with a reduction in STAT6-phosphorylation. This reduction upgraded the effect of 5-FU by increasing both levels of apoptosis and markers of cell adhesion such as E-cadherin, whereas decreased epithelial–mesenchymal transition markers were associated with aggressive phenotypes and drug resistance, such as β-catenin nuclear translocation and Zinc finger protein SNAI1 (SNAI1). Additionally, *Il-10, Tgf-β*, and *Il-17a*, critical pro-tumorigenic cytokines, were downmodulated in the colon by these adjuvant therapies. In vitro assays on human colon cancer cells showed that Trimethylglycine also reduced STAT6-phosphorylation. Our study is relatively unique in focusing on the effects of the combined administration of AS1517499 and Trimethylglycine together with 5-FU on already established CAC which synergizes to markedly reduce the colon tumor load. Together, these data point to STAT6 as a valuable target for adjuvant therapy in colon cancer.

## 1. Introduction

Colon cancer is the second most common cancer in women and the third most common cancer in men worldwide [1]. Colorectal cancer (CRC) has a diverse range of risk factors, with chronic intestinal inflammation associated with ulcerative colitis and Crohn’s disease being a notably important factor associated with colon cancer development [2]. When the inflammation becomes chronic and persists, as is the case in these diseases, it generates an environment that exceeds the mechanisms of immune surveillance and creates a microenvironment that favors the inhibition of the antitumor immune responses, thus favoring the establishment and growth of cancer cells and colitis-associated colon cancer (CAC) [3]. Colon cancer is generally difficult to treat and induces high mortality rates [4]. One of the great challenges that faces clinicians in the treatment of this neoplasia it is during the clinical stage in which the cancer is detected. Unfortunately, mostly patients with CAC are diagnosed in advanced stages, making successful treatments more complicated, leading to a necessity to use broad-spectrum chemotherapeutic agents such as 5-fluorouracil (5-FU) and oxalaplatin (OXP or L-OHP), which are the drugs more frequently utilized in the treatment of colon cancer [5]. Despite efforts, a significant proportion of patients eventually develop resistance to these drugs, limiting its clinical usefulness [6,7]. The resistance acquired by the tumoral cells could be mediated by intrinsic or extrinsic factors, and it is well established that the tumor-microenvironment regulation of inflammation has been implicated in the acquisition of chemotherapeutic resistance [8]. Non-steroidal anti-inflammatory drugs are a viable alternative for reducing the risk of CAC development, which suggests that mechanisms regulating the inflammation play a primary role in the development and treatment of this neoplasia [9].

Trimethylglycine (betaine) is an essential osmoprotectant that was recently reported to be beneficial in several human diseases that have a strong inflammatory component [10]. In nasopharyngeal carcinoma, betaine intake was associated with a reduced risk of development of this neoplasia. In colorectal cancer metanalysis it was observed that high levels of Trimethylglycine were associated with a reduced risk of cancer incidence, suggesting that use of this osmoprotectant and anti-inflammatory agent may function as a preventive treatment in colon cancer [11]; however, experimental support is missing for this idea in both established and early stages of colon cancer development.

The STAT6 protein is highly involved in the regulation of inflammatory processes associated with carcinogenesis [12]. This protein is activated by phosphorylation of the Janus kinase (JAK) family proteins in response to the binding of interleukin (IL) 4 or IL-13 to their common type II IL-4 receptor. STAT6 has a dual function, as it is capable of signaling and transcription, through the dimerization of phosphorylated STAT6 followed by its subsequent translocation to the nucleus, which turns on different genes [13]. The signaling pathway initiated by the phosphorylation of this protein plays an important role in a diversity of cell types, including immune cells and cancer cells [13,14]. STAT6 activation participates in the malignancy of different types of cells, including colon cancer cells, prostate, breast, and mediastinal large B-cell lymphoma [15]. STAT6 knockout (STAT6^−/−^) mice exhibit defective Th2 cell development associated with strong TH1-type responses [16]. In primary and metastatic mammary carcinomas, the deficiency of STAT6 correlates with enhanced tumor immunity and the faster rejection of implanted tumors [17,18]. 

In two recent reports, it has been found that STAT6 protein plays a fundamental role in early colon cancer development, in which STAT6^−/−^ mice decreased tumor growth by 70% [12]. Additionally, at the same time, the capacity of STAT6 was reported to promote intestinal tumorigenesis by expansion of myeloid-derived suppressor cells (MDSCs) [19]. Moreover, in glioblastomas the function of STAT6 has been related with survival and drug resistance, and may be an attractive candidate for targeted therapies [20]. However, the direct participation of STAT6 in the treatment of colon cancer has not been fully established. 

In this study, we have examined whether modulating STAT6 activity and favoring an anti-inflammatory microenvironment with Trimethylglycine may enhance the response to 5-FU in already established colitis-associated colon cancer (CAC). We identified that the inhibition of STAT6-phosphorylation through the use of the inhibitor AS1517499 has a prominent adjuvant effect when used together with the 5-FU drug. Additionally, we tested the use of Trimethylglycine as a possible anti-inflammatory-adjuvant and found that Trimethylglycine was able to inhibit STAT6 phosphorylation, which significantly enhanced the effect of 5-FU treatment leading to the reduction in the number of tumors. 

## 2. Results

### 2.1. STAT6 Inhibitor and Trimethylglycine as Adjuvant Therapies Improve the 5-FU Treatment on CAC

**Figure 1:**
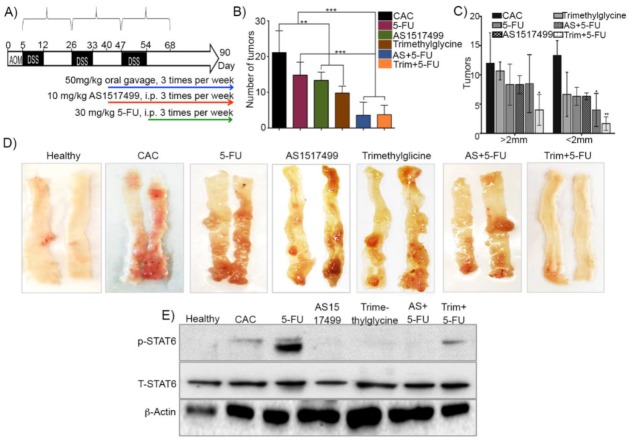
Adjuvant effect of AS1517499 and Trimethylglycine on 5-fluorouracil (5-FU) treatment in colitis-associated colon cancer (CAC). (**A**) Experimental design of the in vivo model to induce CAC. The scheme indicates the days of exposure to Azoxymethane (AOM) and dextran sodium sulfate (DSS) and treatments with AS1517499 and Trimethylglycine in combination with 5-FU and as single therapies. (**B**,**C**) Average number and size of tumors in mice with CAC without treatment (CAC), 5-FU, AS1517499, Trimethylglycine, and combination of AS1517499+5-FU (AS+5-FU) and Trimethylglycine+5-FU (Trim+5-FU). (**D**) Images of colons opened longitudinally showing macroscopic morphology after CAC induction and treated and non-treated (CAC) with 5-FU, AS1517499, and Trimethylglycine on day 90. (**E**,**F**). Representative Western blot and densitometry analysis of total extracts from colon tissues of mice treated with 5-FU and STAT6 inhibitor, Trimethylglycine, and their combination. In all of them, p-STAT6 levels were normalized relative to the total STAT6 protein levels in CAC without treatments. *p* value lower than 0.001 (***); *p* = 0.01 (**) and *p* = 0.05 (*). Data are representative of two independent experiments. *n* = 4–6 mice per group.

STAT6 phosphorylation plays a critical role in favoring early CAC development [12]. Here we evaluated the role of STAT6 inhibition activity in established colon tumors in order to assay its possible adjuvant effect on 5-FU therapy. AS1517499 and Trimethylglycine were administrated i.p. and via oral gavage respectively. Both therapies were analyzed individually and as adjuvant therapies together with 5-FU. In order to know these effects, the mouse AOM/dextran sodium sulfate (DSS) CAC model was utilized (Figure 1A). First we found that CAC-untreated mice (CAC group) developed more than 20 tumors each in the distal and middle regions of the colon (Figure 1B), whereas mice receiving single therapy with 5-FU since day 54 until day 90 (5-FU group) displayed a reduction of around 28% in the number of tumors (Figure 1B). The administration of AS1517499 and Trimethylglycine was initiated on day 40 after CAC induction and maintained until day 90. Mice treated with the STAT6 inhibitor developed 29% less tumors compared to untreated CAC mice, whereas mice receiving Trimethylglycine displayed a significant reduction of tumors (45% less) than untreated CAC mice. To determine the adjuvant effect of AS1517499 and Trimethylglycine on 5-FU treatment, we first exposed the mice to these compounds and then in combination with 5-FU. Notably, both adjuvant therapies, AS1517499+5-FU (AS+5-FU) and Trimethylglycine+5-FU (Trim+5-FU), displayed a significant reduction of up to 80% of colon tumorigenesis compared to CAC untreated mice and these adjuvant therapies enhanced the effect of 5-FU single therapy. Moreover, these tumors were also smaller in size compared to those of untreated CAC mice and 5-FU-treated mice (Figure 1B–D). 

These data suggest that STAT6 participates in the tumor progression and its inhibition enhances the effect of conventional pharmacological treatment used in CAC. Similarly, Trimethylglycine favored the effect of 5-FU treatment.

#### Trimethylglycine May Regulate STAT6 Phosphorylation

To confirm the biological effect of the inhibition of STAT6 phosphorylation in our CAC mouse model, Western blot assays were carried out to evaluate the levels of this protein. The results shown in Figure 1E indicate that STAT6 phosphorylation was significantly reduced in colon tissue from mice receiving the inhibitor AS1517499 as single therapy as well as adjuvant together with 5-FU (AS+5-FU) compared to untreated CAC mice; while in mice receiving 5-FU the levels of STAT6 phosphorylated were increased (Figure 1E,F). Strikingly, when we analyzed the levels of STAT6 phosphorylation in colon tissue of mice receiving Trimethylglycine as a single therapy, we found a significant reduction on STAT6 phosphorylation at similar levels showed by the well-known AS1517499 pharmacologic inhibitor of STAT6 (Figure 1E,F). This finding suggests that Trimethylglycine may also inhibit STAT6 phosphorylation and in thereby induces 5-FU sensitivity in the CAC model.

### 2.2. Adjuvant Therapies with AS1517499 and Trimethylglycine Enhance E-Cadherin and Modulate β-Catenin Tissue Expression

**Figure 2:**
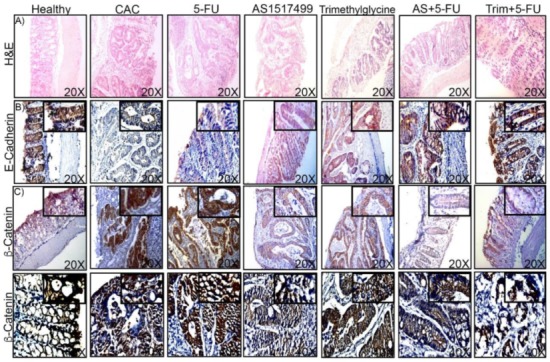
STAT6 inhibition favors recovering of cell adhesion markers in mice treated with 5-FU and decreases β-catenin nuclear localization. (**A**) Representative H&E-stained colonic sections from Healthy tissue (Healthy), CAC without treatment, CAC+5-FU (5-FU), CAC+ AS1517499 (AS), and Trimethylglycine (Trim) as singles therapies and the adjuvant effect with 5-FU, AS+5-FU, and Trim+5-FU. (**B**) Immunohistochemical staining of E-Cadherin and, β-catenin staining (**C**,**D**) in colon tissue obtained from mice with the different treatments previously mentioned. The arrows show the localization of β-catenin in the epithelial cells. The sections of tissue were analyzed with SP8 confocal microscope at 20× and 40×. The magnification was obtained of 40×. Data are representative of two independent experiments with 4–6 mice per group.

Colons from all the groups of mice were sectioned and processed for histological and immunohistochemical analysis. Tissue sections were stained with hematoxylin and eosin (H&E) (Figure 2A). The healthy colon tissue (Control) showed well-defined crypts lined up in parallel, while altered morphology was evident in the tissue sections of CAC untreated mice as well as in mice receiving single therapy with 5-FU, which exhibited glandular adenocarcinomas constituted by atypical epithelial cells with dysplastic nuclei and numerous mitotic figures and chronic inflammation confined to the lamina propria. In the tissue corresponding to AS1517499 and Trimethylglycine as single therapies, the morphology of the intestinal tissue, inflammatory infiltrate, and crypt distortion was observed with less severity in comparison to the CAC untreated mice. The adjuvant therapies for 5-FU, AS+5-FU, and Trim+5-FU, showed a significant reduction in the crypt size and leukocyte infiltrate, indicating a reduction of the inflammatory microenvironment (Figure 2A) as well as less tissue damage, despite the fact some hyperplasia was still observed. Most of the different drugs utilized in cancer are not only intended to reduce the number of tumors, but are also in their capacity used to inhibit tumor progression. In this context, we evaluated the expression of the E-cadherin protein, a well-known cancer progression biomarker [21], in the colon tissues from mice with different treatments. The loss of biomarkers such as E-cadherin, a critical molecule involved in cell adhesion, is one of the main mechanisms underlying cancer invasion and progression [22]. The expression of E-cadherin is characteristic of healthy tissue as observed in the control tissue of colon (Figure 2B), and as expected, immunohistochemical analysis in colon tissue showed that E-cadherin was clearly reduced in untreated CAC mice, whereas 5-FU single therapy did not revert this loss (Figure 2B). Mice treated with AS1517499 and Trimethylglycine as single therapies showed a recuperation of E-cadherin expression (Figure 2B). Interestingly, the use of AS1517499 and Trimethylglycine as adjuvants for 5-FU showed enrichment on E-cadherin on colon tissues (AS+5FU and Trim+5-FU, Figure 2B). Additionally, E-cadherin plays an important role in maintaining epithelial integrity, forming the complex E-cadherin/β-catenin in the adherent junctions [23]. Disassembling of the complex is associated in several types of cancer with more aggressive phenotypes such as epithelial–mesenchymal transition (EMT), as when β-catenin is released it is accumulated in the cytoplasm, and if it is not degraded, a large proportion of it is translocated to the nucleus where it induces expression of its target genes [24]. To determine whether treatment with AS1517499 and Trimethylglycine may inhibit the translocation of β-catenin to the nuclei, we assessed the β-catenin expression in colon tissue. In untreated CAC mice and 5-FU single therapy treated mice the active β-catenin was mainly localized in the nuclei of epithelial cells, while colon tissues obtained of the treatments with AS1517499 and Trimethylglycine showed less β-catenin expression and the localization was more evident in the cytoplasm. In the adjuvant therapies AS+5-FU and Trim+5FU the intensity of nuclear β-catenin labeling was clearly decreased, showing a relocating of β-catenin expression in the cytoplasm and periphery of the epithelial cells (Figure 2C,D).These data reveal the important adjuvant role of Trimethylglycine and AS1517499 in the negative regulation of markers associated with malignant cancer phenotype.

### 2.3. STAT6 Inhibition Increases Apoptosis in Colitis Associated Colon Cancer

**Figure 3:**
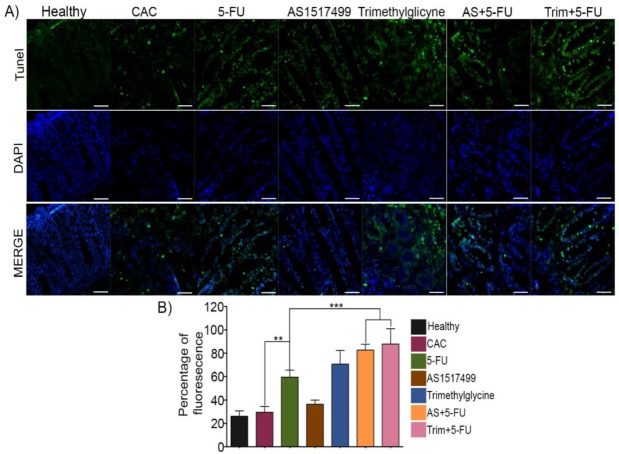
The inhibition of STAT6 increased apoptosis in colitis-associated colon cancer. (**A**) Representative stains of colon tissue in TUNEL assay in CAC treated with STAT6 inhibitors and 5-FU and in combinatory therapies. Green color indicates apoptosis in representative tissue and color blue was DAPI stained for nuclei. (**B**) Fluorescence quantification in colon tissue of TUNEL+ cells. The data are presented as the percentage of mean fluorescence, which was expressed as arbitrary units. Scale bars = 20 μm. The data are expressed as the mean ± SE from 4–6 mice per group representative of two independent experiments. **, *p <* 0.01; ***, *p* < 0.001.

5-fluorouracil is the main chemotherapeutic agent utilized in colorectal cancer, inhibits the metabolism of RNA synthesis with the aim to kill cancerous cells [25]. The response rate associated with 5-FU monotherapy is relatively low (10–15%), but in combination with other therapies, this can be increased to as much as 50% [6,26,27]. In this direction, we evaluated whether STAT6 inhibition using AS1517477 and Trimethylglycine as adjuvant therapies to 5-FU favored the induction of apoptosis of colon cancer cells. Figure 3A shows the DNA damage in the different treatments, which was detected by using terminal deoxynucleotidyl transferase-dT-mediated dUTP nick end labelling (TUNEL) staining, an assay for DNA breaks that uses enzymatic labeling of free 3′DNA ends as one of the features of apoptosis [28]. The treatment of 5-FU as a single therapy displayed a significant increase in the percentage of TUNEL positive cells compared to untreated CAC mice. The combination of AS1517499 and Trimethylglycine together with 5-FU significantly increased the number of TUNEL positive cells by up to 70–80% (Figure 3A,B), indicating that combinatory therapies enhance the apoptosis effect of 5-FU.

### 2.4. STAT6 Inhibition Plus 5-FU Modulates the Expression of Proteins Involved in Apoptosis and Cell Survival

**Figure 4:**
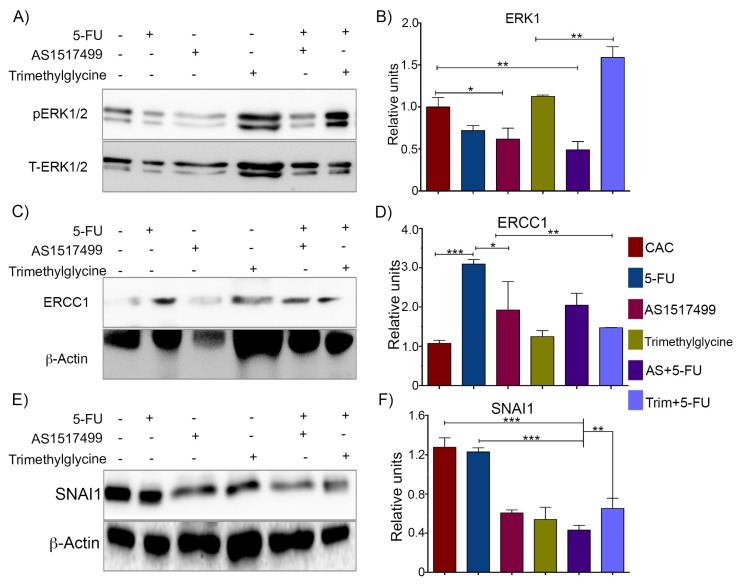
STAT6 inhibition modulates the expression of proteins involved in inhibition apoptosis and cell survival. (**A**,**B**) Representative Western blot and densitometry analysis of ERK1/2 phosphorylation in CAC without treatments, 5-FU, AS1517499, and Trimethylglycine as single therapies and the combination as adjuvants AS+5-FU and Trim+5-FU. (**C**,**D**). Representative Western blot and densitometry analysis of ERCC1 expression under the condition previously mentioned. (**E**,**F**) Representative Western blot and densitometry analysis of SNAI1 expression under the condition previously mentioned. In all of them, the levels of the protein analyzed were normalized relative to the protein levels in CAC without treatments. Densitometry analyses of Western blots show the results of at least two independent experiments with four mice per group. *p* value lower than 0.001 (***); *p* = 0.01 (**) and *p* = 0.05 (*).

The mechanisms involved in the response to 5-FU and additional treatments in colon cancer are diverse. Upregulation of proteins related with DNA repair and inhibition of apoptosis are the most frequent alterations found in cancer with low rates of treatment response [29]. Extracellular signal-regulated kinases 1 and 2 (ERK 1/2), (also known as mitogen activated protein kinases MAPK p44 and p42), regulate cell division, apoptosis, and motility [30]. In vitro studies indicate that phosphorylation of ERK proteins has a negative effect in the response to 5-FU based chemotherapy [31]. Therefore, we investigated whether the phosphorylation of ERK1/2 may be involved in the effect of adjuvant therapies with 5-FU. Figure 4A shows an evident phosphorylation of ERK1/2 in colon proteins from untreated CAC mice, whereas 5-FU single therapy induced a slightly decreased degree of phosphorylation of this protein. The CAC mice receiving AS+5-FU displayed a significant reduction in ERK1/2 phosphorylation (Figure 4A,B). In contrast, mice that received Trim+5-FU showed increased levels of ERK1/2 phosphorylation. Trimethylglycine therapy did not induce ERK1/2 changes compared to CAC untreated mice.

Excision repair cross-complementing group 1 (ERCC1) is another protein associated with the inhibition of apoptosis [32]. In colon cancer ERCC1 expression has been studied as a marker for sensitivity to combined therapies of 5-FU with other drugs, such as cisplatin, and different reports indicate that a high expression of ERCC1 is associated with poor prognosis [33].

Figure 4C,D shows that single 5-FU therapy significantly increased the expression of ERCC1 up to three-fold above the levels observed in untreated CAC mice, whereas the single AS1517499 and Trimethylglycine therapies caused a significant reduction in ERCC1 protein expression. Similarly, the ERCC1 levels displayed in the colon after the adjuvant administration of those therapies with 5-FU were also significantly reduced. These data suggest that ERCC1 could be regulated by STAT6 activity.

Finally, we evaluated SNAI1 expression. SNAI1 is considered a marker of EMT, and it is a factor highly involved in the acquisition of resistance to pharmacological treatment in cancer [34]. High levels of SNAI1 in cancer are associated with downregulation of E-cadherin expression, consequent activation of the Wnt/β-catenin pathway, and the acquisition of an aggressive tumor phenotype [35]. Figure 4E shows a representative Western blot of protein extracts from colon tissues of mice treated with different therapies. The untreated CAC and 5-FU treated mice showed similar levels of SNAI1 expression; while AS1517499 and Trimethylglycine treated groups displayed significantly lower expression of this protein, and the adjuvant therapies with AS+5-FU and Trim+5-FU also revealed a downregulation of SNAI1.

### 2.5. Circulating Monocytic CD11b^+^Ly6C^hi^ and Granulocytic CD11b^+^Ly6C^low^Ly6G^+^Cells Are Modulated by AS1517499 and Trimethylglycine during CAC

**Figure 5:**
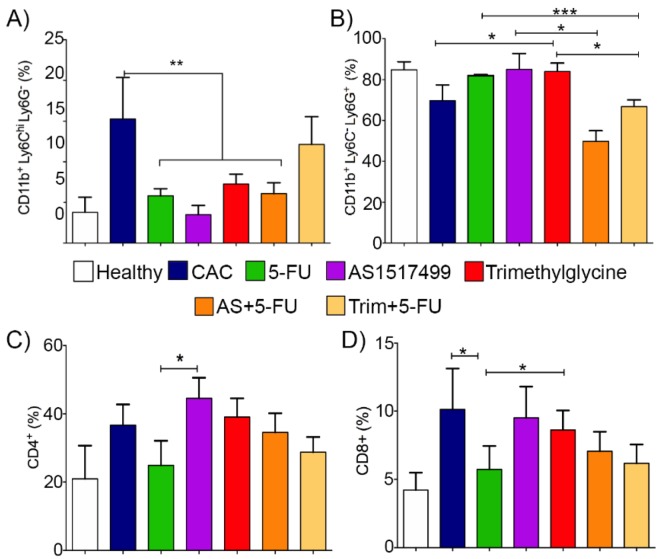
Adjuvant therapy for 5-FU modulates the percentages of circulating inflammatory monocytes and granulocytes in CAC. Flow cytometric analysis was performed with Ly6G and Ly6C markers expressed in the cell surface of circulating cells. (**A**) A representative graph displaying the proportion of CD11b+Ly6C^hi^ monocytes gated on CD11b+ living cells population isolated from the circulation of mice with CAC without treatment, 5-FU, AS1517499, and Trimethylglycine, and a combination of these treatments on day 90 after AOM administration. (**B**) A representative graph displaying CD11b+Ly6ClowLy6G+ cells from the previously mentioned treatments and combinations in animals on day 90 after AOM administration. (**C**) Percentage of CD4+ population cells. (**D**) Percentage of CD8+ cells isolated from the circulation of the mice on day 90 after AOM/DSS administration and treatment with 5-FU and STAT6 inhibitors. The data are representative of two independent experiments that included *n* = 4–6 mice/group. Values are expressed as the mean ± SE. *p* value lower than 0.001 (***); *p* = 0.01 (**) and *p* = 0.05 (*).

To determine the effect of the adjuvant therapies on some immunological cells, we evaluated immune cell populations related with tumor growth in CAC. Myeloid and lymphoid circulating cells were analyzed by flow cytometry. An increase in myeloid monocytic cells in peripheral blood has been associated with some inflammatory pathologies [36]. In particular, CD11b+Ly6C^hi^ (Ly6C^hi^) monocytes increase in number during intestinal inflammation associated with tumor growth and invasiveness [37]. Figure 5A shows a significant increase of CD11b^+^Ly6C^hi^Ly6G^−^ cells in the circulation of untreated CAC mice, but this monocytic population was diminished after the 5-FU, AS1517499, Trimethylglycine, and AS+5-FU therapies; whereas Trim+5-FU only displayed a discrete reduction of this cell population. The flow cytometric analysis also revealed that the granulocytic CD11b^+^Ly6C^−^Ly6G^+^ cell population was enhanced by the Trimethylglycine single treatment, but was not in combination with 5-FU. Interestingly, the AS+5-FU and Trim+5-FU therapies promoted a significant reduction in the CD11b^+^Ly6C^−^Ly6G^+^ cell population (Figure 5B).

It is widely known that CD8^+^ and CD4^+^ T cells participate in an anti-tumor immune response that could be capable of eliminating tumors even those at advanced stages [38]. Next, we evaluated the levels of circulating CD8^+^ and CD4^+^ cells as another alternative of immunological regulation of tumor reduction. Figure 5C indicates that circulating CD4^+^ cells demonstrated a slight difference between the groups 5-FU and AS1517499. In contrast, circulating CD8^+^ T cells were decreased in both 5-FU single treatment and Trim+5-FU adjuvant therapy (Figure 5D).

### 2.6. Pro-Tumorigenic Cytokines Are Regulated by the Adjuvant Use of AS1517499 and Trimethylglycine in Colitis Associated Colon Cancer

**Figure 6:**
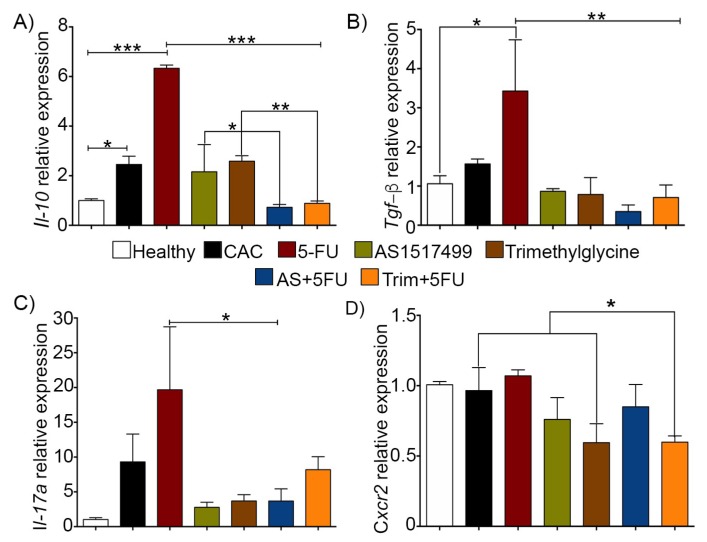
Pro-tumorigenic cytokines are regulated by the adjuvants AS1517499 and Trimethylglycine in CAC. (**A**–**D**). Relative expression of *Il-10, Tgf-β, Il-17a,* and *Cxcr2* genes determined by qPCR in CAC without treatment (black bars), AS1517499 (red bars), Trimethylglycine (green bars), as single therapies and AS1517499+5-FU (AS+5-FU) (brown bars), and Trimethyglycine+5-FU (Trim+5-FU) (orange bars); all relative expressions were compared with the expression levels in healthy tissues (white bars). Data are presented as individual values with median and range from two independent experiments performed in triplicate. *p* value lower than 0.001 (***); *p* = 0.01 (**) and *p* = 0.05 (*).

A primary component of the tumor microenvironment are the cytokines that participate in immunoregulation, and which play a role in different inflammatory processes [3]. In this context, we evaluated the levels of mRNA expression of *Il-10*, *Tgf-β* and *Il-17a*. *Cxcr2* was also analyzed, given that it plays a role in the tumor microenvironment by recruiting neutrophils to inflammatory sites [39]. Figure 6A shows that untreated CAC mice displayed an increased expression of *Il-10* mRNA in the colon tissue with tumors, whereas the 5-FU single therapy group showed a significantly higher expression of this cytokine. In contrast, the use of AS1517499 and Trimethylglycine showed a significant reduction in *Il-10* mRNA expression. The use of these therapies as adjuvant for 5-FU, reduced the expression of *Il-10* mRNA compared to the 5-FU single treatment by over 50% (Figure 6A). Single 5-FU therapy also induced high expression of *Tgf-β* and *IL-17a* mRNA in colon tissue, but the use of AS1517499 and Trimethylglycine as adjuvants for 5-FU downregulated the levels of both of these mRNA (Figure 6B,C). Finally, the chemokine receptor *Cxcr2* has been reported as an important recruiter of inflammatory cells, and is associated with poor outcomes for different types cancers [40,41]. We did not find changes between the CAC, 5-FU, and AS1517499 groups (Figure 6D). *Cxcr2* expression was downregulated by Trimethylglycine alone and in combination with 5-FU, where a significant decrease of *Cxcr2* was observed compared to untreated CAC and 5-FU groups (Figure 6D).

These data suggest that use of AS1517499 and Trimethylglycine not only regulates STAT6 activity but induce regulation in the cytokines related with the tumor microenvironment and tumor progression.

### 2.7. Trimethylglycine Decreases STAT6 Phosphorylation in Human Epithelial Colon Cancer Cells

**Figure 7:**
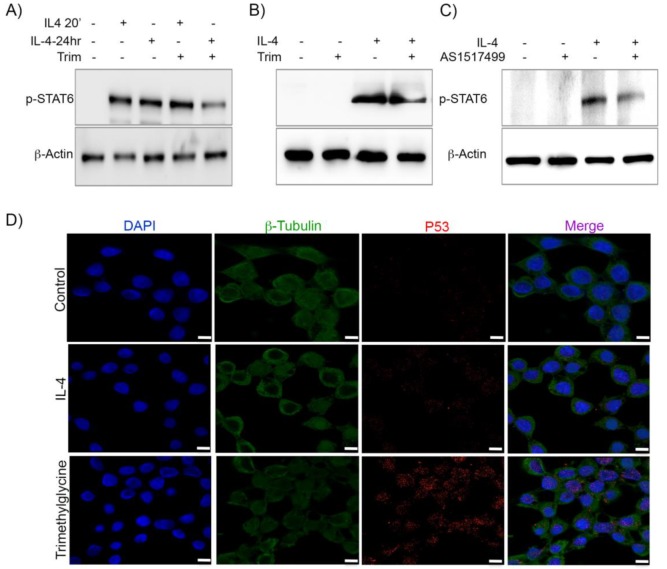
Trimethylglycine decreases STAT6 phosphorylation in a human epithelial colon cancer cell line. (**A**–**C**) Representative Western blots showing the protein levels of phosphorylated STAT6 in HCT-116 colonic human cells. (**A**). Epithelial HCT-116 cells were stimulated with recombinant IL-4 for 20 min and 24 h with and without Trimethylglycine (IL-4-20′+Trim and IL-4-24 h+Trim). (**B**) Representative Western blot showing the levels STAT6 phosphorylation with the stimulus of Trimethylglycine, IL-4, and Trimethylglycine plus IL-4 at 24h. (**C**). Representative Western blot showing the levels of STAT6 phosphorylation using the STAT6 inhibitor AS1517499 (300mM). (**D**). Representative immunofluorescence assay for P53 protein on HCT-116 cells exposed to IL-4 and Trimethylglycine. Alexa 488 (green) color indicates the expression of P53 in these cells and DAPI (blue) was used to visualize nuclei; scale bars = 20 μm. Confocal analyses were performed using the Leica TCS SP8 confocal microscopy system.

In order to explore further the mechanism associated with the remarkable effect observed by the Trimethylglycine therapy, we analyzed the inhibition of STAT6 phosphorylation in epithelial cells by using HCT-116 human colonic cancer cells. This cell line does not have a constitutive phosphorylated STAT6 phenotype. First, we induced the STAT6 phosphorylation through IL-4 stimulation; this activation was evaluated after exposure to Trimethylglycine for 20 min and 24 h. Figure 7A shows that, in HCT-116 cells exposed to IL-4, the STAT6 activation was evident since 20 min with equal levels of expression at 24 h. The assay to evaluate the effect of Trimethylglycine on HCT-116 cells was performed in the same conditions for 24 h in the presence of IL-4 stimulus. The result in Figure 7A,B showed that STAT6 phosphorylation under Trimethylglycine treatment for 24 h decreased by around 50% compared to IL-4 stimulation alone (Figure 7B). At the same time, similar conditions were carried out on an assay with the well-known STAT6 inhibitor AS1517499. Figure 7C shows representative Western blot from the extracts of HCT-116 cells stimulated with IL-4 and exposed to AS1517499, where a reduction up to 60% of STAT6 phosphorylation was detected. Additionally, it has been reported in cervical cancer that Trimethylglycine is able to increase the levels of P53 protein [42], thus, in order to determine whether a similar effect occurred in our system, we performed immunofluorescence assays on HCT-116 cells and detected a high expression of P53 in cells exposed to Trimethylglycine in comparison to the control and IL-4 stimulated cells (Figure 7D). Together these findings confirm that Trimethylglycine is able to inhibit STAT6 phosphorylation and favor P53 expression.

## 3. Discussion

Colorectal cancer (CRC), independent of its origin, is frequently diagnosed at advanced stages, is generally difficult to treat, and generates high mortality rates [4]. The influence of the inflammatory microenvironment in the response to treatments in cancer has acquired great relevance in the last few years [8]. 5-fluorouracil (5-FU) is the main chemotherapeutic agent utilized in the treatment for CRC, however, the response rate associated with 5-FU monotherapy is relatively low (10–15%), but its combination with other agents such as oxaliplatin and irinotecan can improve the response rate up to 50% [6,26,27]. Nonetheless, the negative side effects of drug combinations lead to nausea, vomiting, hematologic toxicity, and chronic renal insufficiency [43,44]. For these reasons, a constant search for new therapeutic strategies to improve the response rates of conventional therapies and to reduce the adverse side effects of such therapies is necessary in CRC.

In the present study, we found that in the in vivo AOM/DSS CAC model, the inhibition of STAT6 phosphorylation by AS1517499 and the use of Trimethylglycine induced a remarkable reduction in tumor growth when they were administrated together with 5-FU. Therefore, these compounds may be considered as potential adjuvant therapies to improve 5-FU effectivity during CAC development.

The AS1517499 is a well-known potent and selective STAT6 inhibitor [45], and we proved here the critical role of STAT6 pathway, not only in the progression and development of cancer as previously reported [12], but also its synergistic activity when combined with 5-FU therapy in the already established colon cancer.

Trimethylglycine (betaine) is a derivative of the amino acid glycine, a non-toxic natural substance; its physiological roles include osmoprotectant and methyl group donor activities. In addition, several reports found that Trimethylglycine has an anti-inflammatory effect on different diseases [10,11]. Particularly, in cancer it has been reported that Trimethylglycine intake may reduce the risk of nasopharyngeal carcinoma, breast, lung, liver, and colorectal cancer, highlighting a possible protective effect in the inhibition of tumor development [10,46], but the specific effect of its treatment has not yet been established. Here we showed that the use of Trimethylglycine has important effects on the development of colon tumorigenesis in the CAC model, but its use as a novel adjuvant therapy together with 5-FU enhances the effect of this chemotherapeutic drug. Interestingly, this inhibiting effect on tumor growth could be associated with STAT6 inhibition, as we found that Trimethylglycine was a putative inhibitor of STAT6 phosphorylation in in vivo and also in vitro assays, with similar results to those observed with the use of AS1517499 (specific STAT6 inhibitor). In some cancers, the activation of STAT6 is highly related to the increase of apoptosis inhibition, therefore, blocking the phosphorylation of this protein may be a mechanism triggered by Trimethylglycine to improve the response to 5-FU therapy.

Our data showed that the blockade of STAT6 activity used as an adjuvant therapy enhanced the effect of 5-FU by increasing markers of cell adhesion such as E-cadherin. This protein is a central adhesion molecule that acts at the cell–cell adhesion junctions, and the loss of this protein has been associated with the acquisition of malignant phenotypes in various types of human cancers favoring epithelial–mesenchymal transition (EMT), tumor metastasis, and poor clinical outcomes [47]. Yang et al. (2006), reported that colorectal cancer cell resistance to oxaliplatin downregulated E-cadherin expression [48]. Here, we showed that both AS1517499 and Trimethylglycine induced the upregulation of E-cadherin protein levels in colon tissues of CAC mice. The expression of E-cadherin was significantly reduced and showed a close relationship to β-catenin nuclear localization in the tissues of CAC-untreated mice and 5-FU single therapy-treated mice. In agreement with our data, similar results on E-cadherin loss expression and translocation to the nucleus of β-catenin have been reported in breast and pancreatic cancer cells, and this is considered to be a marker of poor survival and resistance to therapies [24,49]. Therefore, these data support our proposal that STAT6 inhibition favors the response to 5-FU by regulating proteins related with cell survival, apoptosis inhibition, and acquisition of aggressive phenotypes in CAC.

The loss of E-cadherin and consequent β-catenin nuclear translocation is a well-known mechanism that increases the expression of diverse proteins such as c-MYC, BCL2, and inhibitors of apoptosis proteins (IAPS) related with the inhibition of apoptosis [24,50]. The use of AS1517499 and Trimethylglycine in the CAC model indicates a direct participation of STAT6 in the chemosensitivity to 5-FU by the induction of apoptosis, pointing to its importance as a possible use as adjuvant therapy to enhance the effectiveness of 5-FU on established CAC. Our data agree with previous results in colorectal cancer, where it was shown that high levels of STAT6 phosphorylation lead to apoptosis resistance when compared to STAT6-defective cell phenotypes [51].

Phosphorylation of ERK1/2 has been reported to activate pro-survival pathways inducing anti-apoptotic proteins such as BCL2, and downregulation of p53 in breast, cervical, and hepatocellular carcinoma [30,52]. Interestingly, we found decreased ERK1/2 phosphorylation mainly in the 5-FU treatment and AS+5-FU, as well as an unexpected increase in the use of Trimethylglycine, which may indicate that administration of this adjuvant favors the ERK1/2 activity as reported in the primary cultures of human osteoblasts [53]; this, however, contends with data reported in lipid metabolism in which the use of Trimethylglycine decreased ERK1/2 phosphorylation [54].

These controversial results suggest the need for more detailed studies to elucidate the mechanisms triggered by Trimethylglycine in the regulation of ERK1/2 in colon cancer.

However, at present, we cannot rule out that an increase in ERK1/2 phosphorylation by Trimethylglycine treatment could be a compensatory or alternative mechanism to escape cell death induced by adjuvant therapy with 5-FU. We showed in our in vitro model of human colon cancer cells that exposure to Trimethylglycine partially inhibits STAT6 phosphorylation that leads to high expression of P53 protein, which may be an additional marker of apoptosis induced by Trimethylglycine [42].

Additionally, we also showed that STAT6 inhibition correlated with the regulation of ERCC1, a protein involved in nucleotide excision repair (NER) induced by DNA damage [33]. In colorectal cancer the expression of ERCC1 has been associated with poor survival and poor response to 5-FU and oxaliplatin combination chemotherapy in metastatic CRC patients [55]. Therefore, the combination of therapies that favor the response to 5-FU and decreased levels of ERCC1 could be considered as a new target in the clinical field. In this context, we observed decreased levels of ERCC1 protein in the colons of mice receiving either AS1517499 or Trimethylglycine adjuvant treatment together with 5-FU. Notably, a possible interaction between STAT6 and ERCC1 has not yet been described, however, it is possible that the expression of a protein that controls survival and apoptosis inhibition such as STAT6, may regulate indirect pathways involved in DNA repair. In the tunnel assay performed on colon tissue we found that AS+5-FU and Trim+5-FU- treated mice displayed greater levels of apoptosis; we correlated this effect with a decreased expression of a protein related to apoptosis inhibition such as BCL2, which is diminished in the absence of STAT6 [12].

Moreover, some reports have indicated that STAT6 overexpression plays a critical role in promoting EMT and aggressiveness of CRC through Zinc-finger E-box binding homeobox 1 (ZEB1) activation, which is currently considered as a factor associated with chemoresistance [56]. In line with this, we found that STAT6 regulates SNAI1, another typical marker of EMT, which has an important role in E-cadherin expression and that the levels of SNAI1 were inversely related with E-cadherin expression, as reported in esophageal cancer [47]. Thus, our studies provided information about a possible mechanism in which STAT6 inhibition favored the response to 5-FU in CAC by regulating markers of EMT and proteins related with cell survival and inhibition of apoptosis.

The response to therapy in many tumors including those associated with colorectal cancer is mediated by diverse mechanisms. The tumor surrounding non-malignant cells, such as stromal cells, including fibroblast, immune cells, and cells from the vasculature, can be determinant in this response [57].

STAT6 is an important factor in the activation of the monocyte population that participates in cancer development and progression [12]. The monocytes appear to be recruited throughout tumor progression, including during the early stages of tumor growth where they differentiate into macrophages. Previous reports indicate that STAT6 plays a role in macrophage polarization that could lead to carcinogenesis and cancer progression [58]. We evaluated the circulating CD11b^+^Ly6C^hi^ monocytic population in our assays, and AS+5-FU therapy induced a decreased percentage of CD11b^+^Ly6C^hi^ circulating cells, indicating that STAT6 inhibition is a possible target for decreasing an immunological population that may favor resistance to 5-FU. Studies on pancreatic ductal adenocarcinoma indicated that decreased numbers of Ly6C^hi^ monocytes may sensitize tumors to chemotherapy-induced cell death, given STAT6 activity is a requirement for the recruitment of inflammatory Ly6C^hi^ monocytes and their conversion to tissue-remodeling M2 macrophages [59,60]; thus, the reduced expression of STAT6 phosphorylation could be responsible for the reduction of CD11b^+^Ly6C^hi^ cells in our model. On the other hand, CD11b^+^Ly6C^low^Ly6G^+^ granulocytic cells showed a decrease in the adjuvant therapies in the AS+5-FU group, in contrast to those levels detected in treatments such as individual therapies. Ly6G^+^ cells are able to secrete various cytokines and chemokines that promote tumor growth, metastasis, angiogenesis, and suppression of anti-tumor immunity [61]. Thus, our data suggest that inhibition of STAT6 phosphorylation may favor the response to 5-FU by modulating some immune cells such as the CD11b^+^Ly6G^+^ population. Supporting our hypothesis, a recent report has described that fewer levels of circulating Ly6G^+^ granulocytic cells are associated with an improvement of antitumor responses in STAT6^−/−^ mice [12]. In breast cancer cells, the depletion of STAT6 demonstrated that this protein participates in the negative regulation of CD8+ T cells, which are essential for tumor rejection [62]. Nevertheless, we observed a decrease in CD8+ cells in the CAC mice treated with 5-FU, and a slight increase in these for therapies with AS1517499 and Trimethylglycine; however, these results do not discard the possible effect of CD8+ cells in the tumor site, and their possible participation in tumor rejection observed in the mice treated with AS+5-FU. The use of different therapeutic drugs against cancer has been reported to induce changes in peripheral blood T-cells [63]. Hence, more detailed experiments to understand these phenomena using AS1517499 and Trimethylglycine are necessary.

Cytokines are key components in the tumor microenvironment, and could be involved in the response to chemotherapies in CAC during STAT6-inhibition. We observed that *Il-10* expression was significantly reduced in CAC mice treated with STAT6 inhibitors as single therapies and as adjuvants to 5-FU.

High levels of IL-10 have been reported to be responsible for drug resistance to breast cancer, in which the blockade of this cytokine resulted in diminished STAT3 activation and a decrease in *Bcl*-2 mRNA expression, and consequently the induction of apoptosis [64]. The regulation of IL-10 correlates with our results, which indicate that the treatment with AS1517499 and Trimethylglycine as adjuvant therapies with 5-FU had better results in reducing the number of tumors and increasing apoptosis.

The expression of IL-10 could be regulated by T regulatory cells (Tregs), which induce tolerance and limit autoimmune and inflammatory responses by IL-10 and TGF-β production [65]. These cytokines are closely related and are involved in a positive feedback loop, in which IL-10 favors the expression of TGF-β and vice versa [66], whereby the levels of TGF-β were dramatically decreased in the adjuvant therapies in CAC at the same levels of IL-10.

TGF-β causes the differentiation of naive T cells into Tregs, and the presence of both TGF-β and IL-6 promotes the differentiation of Th17 cells. The stimulation with TGF-β and IL-6 leads to CD8+ cell cytotoxic function loss, and consequently induces IL-17 production [67]. Here, we showed that STAT6 inhibition induced downregulation of *Il-17a*, although its well known that levels of IL-17A are not directly regulated by STAT6, decreased *Il-17a* expression could be explained as the indirect mechanism triggered by STAT6 over TGF-β regulation and as a consequence its effect on *Il-17a*.

All these findings demonstrate, for the first time, that Trimethylglycine is a possible inhibitor of STAT6 phosphorylation. Furthermore, the use of AS1517499 and Trimethylglycine as adjuvant therapies enhanced the activity of 5-FU in controlling colon cancer development through a decreased expression of markers associated with the acquisition of aggressive phenotypes such as β-catenin nuclear localization, and a remarkable reduction in the expression of SNAI1. Whereas, increasing E-cadherin expression was observed in mice receiving both adjuvant therapies (AS+5-FU and Trim+5-FU) compared to single 5-FU therapy, and in untreated CAC mice. Our study is relatively unique as it focuses on the effects of combined administration of anti-inflammatory drugs such as AS1517499 and Trimethylglycine together with 5-FU in already established CAC, where such combinations synergize to markedly reduce the colon tumor load. Therefore, overall, these data point to STAT6 being a valuable target for adjuvant therapy in the treatment of an already established colon cancer triggered by a strong inflammatory component.

## 4. Materials and Methods

### 4.1. Mice

Eight-week-old female BALB/c mice were originally purchased from Harlan Laboratories (México City, México) and maintained in a pathogen-free environment at the FES-Iztacala, Universidad Nacional Autonoma de Mexico (UNAM) animal facilities. The animals were fed Purina Diet 5015 and water ad libitum. All experimental procedures were in strict accordance with the recommendations in the Guide for the Care and Use of Laboratory Animals of the National Institutes of Health (USA), and were approved by the Committee on the Ethics of Animal Experiments of the FES-I (UNAM), under number CE/FESI/042017/1168 (18/04/2017).

### 4.2. Induction of Colitis-Associated Colorectal Cancer

The CAC model was carried out as described previously [68]. Briefly, BALB/c mice received an intraperitoneal (i.p.) injection containing 12.5 mg/kg azoxymethane (AOM; Sigma Aldrich, St. Louis, MI, USA). Five days later, 2% dextran sodium sulfate (DSS, MW: 35,000–50,000, MP Biomedicals. Irvine, CA, USA) was dissolved in the animals’ drinking water for seven days. Afterwards, the mice received regular drinking water for 14 days. This experimental series was repeated twice. Throughout the experiment, the mice were monitored weekly for body weight, stool consistency, and the presence of blood in the rectum or stools.

### 4.3. 5-Fluorouracil, AS151749, and Trimethylglycine Treatments in the Mouse CAC Model

At day 40 after AOM injection the treatment with STAT6 inhibitor (AS1517499) and Trimethylglycine were initiated in four different groups of mice. The AS151749 (Axon Medchem, Reston, VA, USA) was reconstituted in dimethyl sulfoxide (DMSO) and PBS. AS151749 (10 mg/kg) was intraperitoneally administrated every third day until the end of the experiments.

Trimethylglycine (Sigma Aldrich) was administrated to the mice with CAC by oral gavage three times per week at 50 mg/kg. Fourteen days after AS1517499 and Trimethylglycine treatment started, a group of each treatment received additional (co-treatment) i.p. injections of 5-FU (Sigma Aldrich). At the same time, the previously untreated CAC group of mice received 5-FU as individual therapy. All the mice were euthanized on day 90 after AOM injection; the colons were removed, weighed, and processed for macroscopic inspection and histopathological examination. Immediately, the colonic tissue was either fixed in 100% ethanol and embedded in paraffin for histopathologic analysis or snap frozen in liquid nitrogen for RNA and protein extraction.

### 4.4. Histologic Analysis

Collected tissues from mice with CAC receiving AS1517499, 5-FU, Trymethyglycine, AS1517499+5-FU, and Trymetilglycine+5-FU, respectively, the untreated group with CAC (CAC), and finally the tissue obtained from healthy mice (Control), were fixed in 100% ethanol and embedded in paraffin for posterior 4-mm cross-sectioning. The tissue sections were stained with hematoxylin and eosin (H&E; for pathologic evaluation).

For immunohistochemical analysis, the sections were deparaffinized in xylene and then rehydrated with graded alcohols and processed as reported previously [69]. The sections were incubated overnight at 4 °C with the respective primary antibodies diluted in 1× PBS (anti-E-cadherin, 1:300, cell signaling; anti-β-catenin, 1:200, GeneTex, Irvine, CA, USA) and then developed following the conventional technique. The slides were analyzed using an AxioVert.A1 image capture optical microscope (Carl Zeiss Microscopy GmbH, Oberkochen, Germany)Tissue microphotographs were captured using an AxioCam MRc and ZEN lite 2011 software v.1.0.1.0 (Oberkochen, Germany). Quantification of E-Cadherin and β-catenin was performed using ImageJ software v.4.9 by counting cells in 10 high-powered fields from each mouse.

### 4.5. Cell Culture

The HT-116 cells (ATCC, Manassas, VA, USA) were cultured in McCoy’s 5A medium (Gibco, Grand Island, NY, USA), supplemented with 10% fetal bovine serum, 50 U/mL penicillin, and 50 μg/mL streptomycin. Cultures were incubated at 37 °C with 5% CO_2_. For experiments, 2.5 × 10^3^ cells/cm^2^ were plated and cultured for 24 h. After switching to McCoy’s 5A medium with 1% FBS for 18 h, cells were cultured without serum and stimulated with 2.5 µM to Trimethylglycine for 24 h. Next, 20 ng/mL of human recombinant IL-4 (Peprotech, Rocky Hill, NJ, USA) for 24 h to trigger STAT6 phosphorylation. The AS1517499 (STAT6 inhibitor) was added 1 h prior to the stimulus with IL-4 at the concentration of 300 mM. Control cells were cultured in the same manner but without IL-4, Trimethylglycine, or AS1517499.

### 4.6. Gene Expression

Total RNA was extracted from the from colon tissue using the DNA/RNA/PROTEIN Kit purification plus (NORGEN BioTek Corp., Thorold, ON, Canada) following the manufacturer’s instructions. The RNA concentration was determined by measuring the absorbance at 260 nm. One microgram of RNA was used for first-strand cDNA synthesis with Revert Aid H Minus First Strand cDNA Synthesis Kit (Thermo Fisher Scientific, Waltham, MA, USA). Gene expression was carried out using the Fast Start SYBR Green Master kit (Applied Biosystems, Foster City, CA, USA), using a 7500 Real-Time Thermal Cycler (Applied Biosystems). Relative gene expression values were normalized to the constitutive expression of the *RPLP0* gene. Specific primers for target genes are shown in Supplementary Table S1.

### 4.7. SDS-PAGE and Western Blotting

Total proteins were obtained from tissue lysates with the RNA/Protein purification Kit (NorBiotek Canada, Thorold, ON, Canada). Additionally, protein extract from cell line were obtained by the RIPA 1× buffer supplemented with Complete^®^ inhibitors cocktail (Roche Applied Science, Mannheim, Germany). Proteins were separated by 10% SDS-PAGE, blotted onto nitrocellulose membranes, and blocked. The membranes were then exposed to mouse anti-Phospho STAT6 (1:1000) or anti-Total-STAT6 (1:1000), anti-SNAI1, anti-ERCC1, or anti-phospho-ERK1/2 and anti-Total ERK 1/2 antibodies (all from Cell Signaling Technology, Danvers, MA, USA). The anti-β-actin monoclonal antibody (Santa Cruz Biotechnology, St. Cruz, CA, USA) was utilized to detect cell actin as the protein load control. Horseradish peroxidase (HRP)-tagged secondary anti-rabbit or anti-mouse antibodies (1:5000) (Jackson Immunoresearch, West Grove, PA, USA) were used for chemiluminescent detection with the ImmobilonTM Western Chemiluminescent HRP Substrate kit (Millipore, Burlington, MA, USA). Chemiluminescence signals were recorded on Alliance Q9 UVITEC imaging device (Cambridge, UK) and densitometric analyses were performed with ImageJ software.

### 4.8. TUNEL Staining

Apoptosis was detected using the In Situ Cell Death Detection Fluorescein Kit (Roche, Basilea, Swiss), analyzing samples with a ZeissVert A1 conventional epifluorescence microscope and a LEICA TCS SP2 confocal microscope (the analyzed area in each sample was 2.8 mm^2^, and 20 fields of 50 mm^2^ were evaluated).

### 4.9. Flow Cytometry

For flow cytometry circulating blood was obtained during animal necropsy. The cells were washed with 1× PBS and blocked using antibodies to CD16/CD32. The cells were simultaneously stained with antibodies to CD11b, Ly6C, Ly6G, CD4, CD8 (BioLegend) for 30 min at 4 °C. The cells were washed twice and analyzed using the FACS Aria Fusion system and Cell Quest software (Becton Dickinson, México City, México).

### 4.10. Statistical Analysis

Data were analyzed either by one-way analysis of variance followed by Tukey’s multiple comparison test or by unpaired two-tailed *t*-tests with GraphPad Prism 5 (San Diego, CA, USA). All tests were performed using 95% confidence intervals. Data are expressed as means ± SE, where * represents *p* < 0.05 and ** represents *p* < 0.01.

## Supplementary Materials

Supplementary Materials can be found at https://www.mdpi.com/1422-0067/21/6/2130/s1.

## Abbreviations

STAT6	Signal transducer and activator of transcription 6
5-FU	5-fluorouracil
EMT	Epithelial–mesenchymal transition
CAC	Colitis-associated colon cancer
AOM	Azoxymethane
DSS	Dextran sodium sulfate
SNAI1	Zinc finger protein SNAI1
ERCC1	Excision repair 1, endonuclease non-catalytic subunit
