# Supplementary material for: Use of STAT6 Phosphorylation Inhibitor and Trimethylglycine as New Adjuvant Therapies for 5-Fluorouracil in Colitis-Associated Tumorigenesis
Source: Int J Mol Sci. 2020 Mar 20;21(6):2130. doi: 10.3390/ijms21062130 (PMC7139326; doi:10.3390/ijms21062130)
Supplement: Supplementary file 1 [file ijms-21-02130-s001.pdf]

Supplementary Table S1. Primer sequences for qPCR analysis.

Gene	Sequence	Melting Temperature
IL-10	F-GCTCTTACTGACTGGCATGAG<br>R-CGCAGCTCTAGGAGCATGTG	60 °C
CXCR2	F-AGCAAACACCTCTACTACCCTCTA<br>R-GGGCTGCATCAATTCAAATACCA	58 °C
Tgf-	F- CTTCAATACGTCAGACATTCGGG<br>R- GTAACGCCAGGAATTGTTGCTA	60 °C
IL-17A	F- TTTAACTCCCTTGGCGCAAAA<br>R- CTTTCCCTCCGCATTGACAC	60 °C
